# Uncommon site of metastasis from renal cell carcinoma: Case report

**DOI:** 10.1016/j.ijscr.2019.02.013

**Published:** 2019-02-13

**Authors:** Mauricio Alves Ribeiro, Caroline Petersen da Costa Ferreira, Bruno de Lucia Hernani, Luiz Arnaldo Szutan, Maria Carolina Galli Mortati, Fabiana Toledo Bueno Pereira, Fabio Kater

**Affiliations:** a Department of Surgery, Irmandade da Santa Casa de Misericórdia de São Paulo and Real e Benemérita Associação Portuguesa de Beneficência, Brazil; b Medical Science School of Santa Casa de São Paulo, Brazil; c Department of Pathology, Irmandade da Santa Casa de Misericórdia de São Paulo and Real e Benemérita Associação Portuguesa de Beneficência, Brazil; d Department of Oncology, Real e Benemérita Associação Portuguesa de Beneficência, Brazil

**Keywords:** Cm, centimeters, CT, computed tomography, EUS, endoscopic ultrasound, GB, gallbladder, Gy, gray, MRI, Magnetic Resonance Imaging, RCC, renal cell carcinomas, Gallblader tumor, Renal cell carcinomas, Latente metastasis, Case report

## Abstract

**Background:**

Renal cell carcinomas (CCR) account for 90% of renal tumors. Presence of latent distant metastasis is characteristic of RCC and may manifest more than a decade after nephrectomy. Gallbladder (GB) is a rare site of metastasis, with few reports in the literature. A case of metastasis from RCC to GB nine years after initial diagnosis is reported herein.

**Case report:**

Male patient, 74 years, nine years post right radical nephrectomy for grade 2 clear-cell adenocarcinoma, T3BN0M0. During onset, Magnetic Resonance Imaging (MRI) evidenced T2-weighted hypointense and T1-weighted hyperintense lesion, with early and persistente contrast enhancement and exophytic bulging of the underlying outer vesicular margin. T1-weighted hypointense and T2-weighted slightly hyperintense nodular formation was also evidenced in the body portion of the pancreas, with 1.5 × 1.2 cm. The patient was subjected to videolaparoscopic cholecystectomy associated to endoscopic ultrasound (EUS) intraoperatively for investigation of the pancreatic nodule. The anatomopathological examination of the gallbladder was compatible with infiltrating metastasis from clear-cell carcinoma of primary renal site. A solid, hypoechoic, oval nodule with 14 mm was found at EUS, which cytology was suggestive of clear-cell Carcinoma. Because this is an indolent disease with oligometastasis, local ablative treatment with fractionated stereotactic radiation therapy with a dose of 40 Gy was selected. The patient is found with stable disease one year after radiation therapy.

**Conclusion:**

Gallbladder is an unusual site of RCC metastasis. In patients with history of this disease, all vesicular lesions should be given attention, even where the primary tumor has been treated many years before.

## Highlights

• Renal carcinoma represents 1–3% of visceral malignancies.
• Metastases of renal tumors may manifest up to a decade after initial injury.
• Gallbladder represents a rare site of metastatic site, with few reports in the literature.

## 1 Background

Renal cell carcinomas (CCR) account for 1%–3% of all malignant visceral neoplasms and 90% of renal tumors. Its prevalence has increased in the recent years and the presence of latent distant metastasis is characteristic of RCC and may manifest more than a decade after nephrectomy. Clear Cell (CC) RCC is the most common type of renal cancer, accounting for 75% of all primary kidney tumours [1].

Gallbladder (GB) is a rare site of metastasis, with few robust reports in the literature containing clear descriptions of imaging, surgical and anatomopathological parts that add information for its recognition [2,3]. The clinical diagnosis of this entity may be laborious, due to the similar characteristics to benign lesions. We report a case of metastasis from RCC to GB and pancreas nine years after initial diagnosis. The work has been reported in line with the SCARE criteria [4].

## 2 Case presentation

**Fig. 1:**
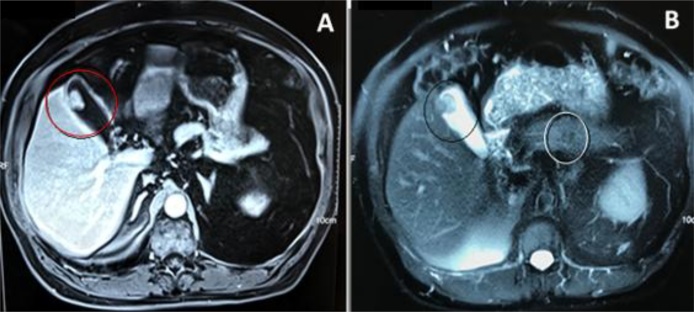
Abdominal MRI. **A**. Expansive formation on the right lateral body wall of the gallbladder, with 1.7 × 1.3 cm, showing pronounced early and persistent contrast enhancement and promoting exophytic bulging of the underlying outer vesicular margin, which shows irregular contours (Red circle). **B**.T2-weighted hypointense expansive formation in the right lateral body wall of the gallbladder (black circle) and T2-weighted slightly hyperintense nodular formation in the body portion of the pancreas (White circle).

**Fig. 2:**
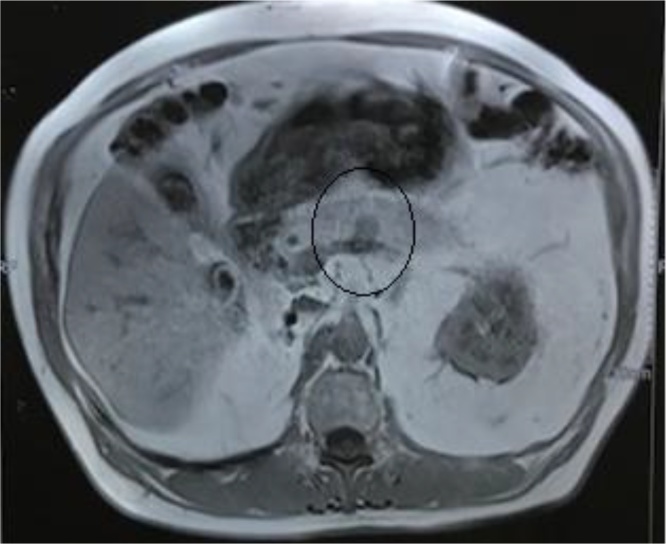
T1-weighted hypointense nodular formation in the body portion of the pancreas with 1.5 × 1.2 cm (circle).

Male patient, 74 years old, nine years post right videolaparoscopic radical nephrectomy for grade 2 clear-cell adenocarcinoma, T3BN0M0 (not subjected to systemic chemotherapy), during annual onset on private practice setting, it was found a gallbladder polyp with 0.7 × 0.7 cm on computed tomography (CT). He was completely asymptomatic. After one year, in 2017, CT was repeated with evidence of polyp growth to 1.7 × 1.3 cm. Investigation was complemented with Magnetic Resonance Imaging (MRI), which evidenced T2-weighted hypointense and T1-weighted hyperintense lesion, with early and persistent contrast enhancement and exophytic bulging of the underlying outer vesicular margin, showing irregular contours (Fig. 1A and B). T1-weighted hypointense and T2-weighted slightly hyperintense nodular formation was also evidenced in the body portion of the pancreas, with 1.5 × 1.2 cm (Fig. 2). Chest CT and bone scintigraphy were also conducted, which showed no secondary lesions in bones and lungs. He had no alteration in laboratory exams (Table 1) [5].

**Table 1:** Laboratory exams before surgery.

Laboratory Exams	Admission values	Reference values
Hemoglobin	15,7 g/dL	14–18 g/dL [3]
Leukocytes	4,46 thousand/uL no deviations	4 thousand – 11 thousand/uL [3]
Lactic deshydrogenase:	429 mg/dL	180–460 U/L* [3]
Creatinine	1,4 mg/dL	0.7–1.5 mg/dL [3]
Urea	40 mg/dL	8–20 mg/dL [3]
Psat	2,96 ng/ml	Until 4,0 ng/ml [3]

One month later, the patient was subjected to videolaparoscopic cholecystectomy associated to endoscopic ultrasound (EUS) intraoperatively for investigation of the pancreatic nodule.

**Fig. 3:**
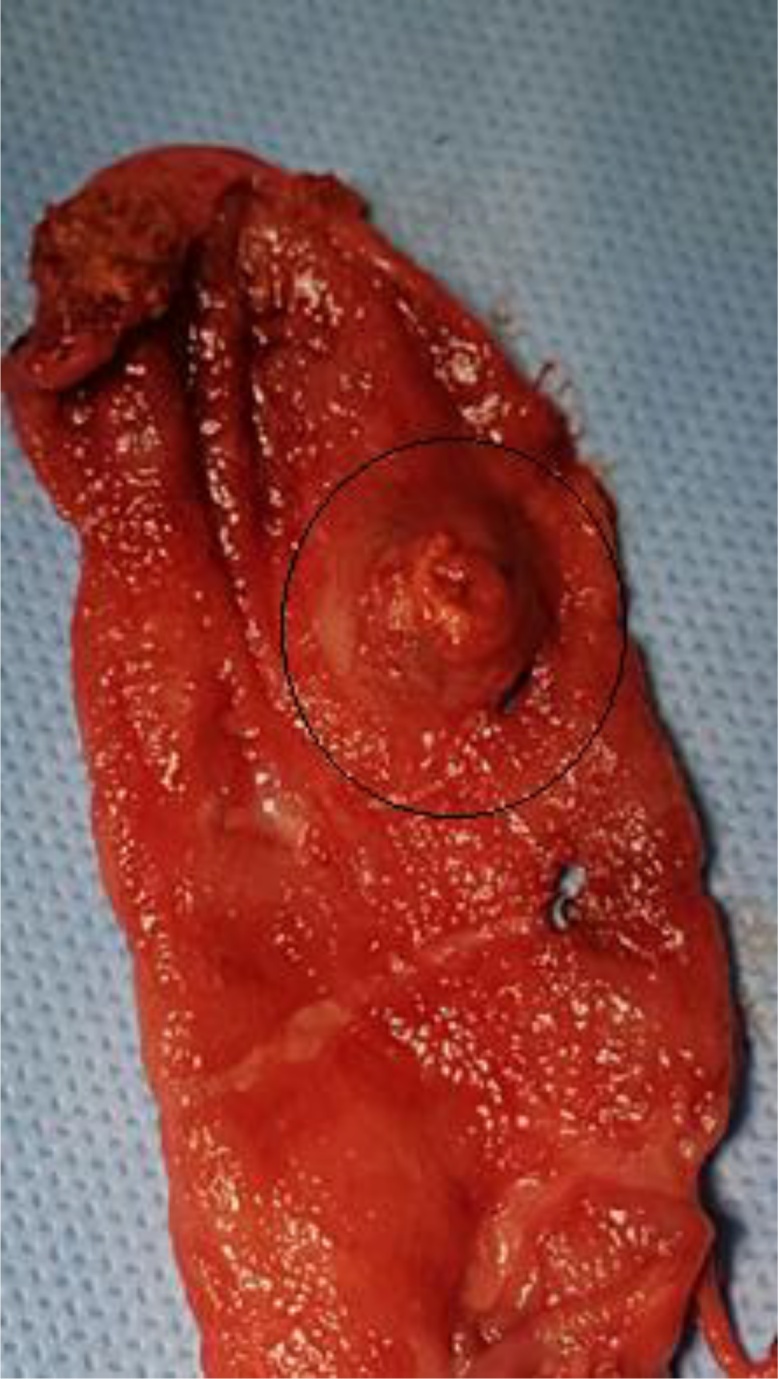
Metastatic renal cell carcinoma as a well-circumscribed polypoid mass in the gallbladder body (circle).

**Fig. 4:**
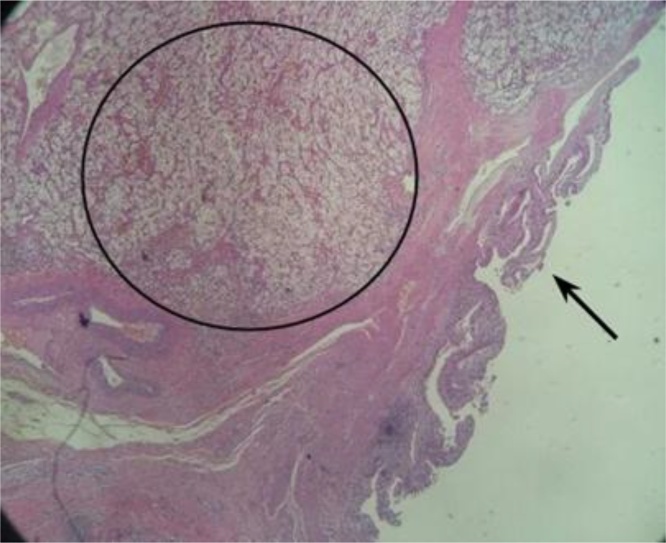
Gallbladder with areas of mucosal erosion (arrow) and metastatic neoplastic process constituted of wide and clear cytoplasm cells, permeating the wall of the organ (circle).

**Fig. 5:**
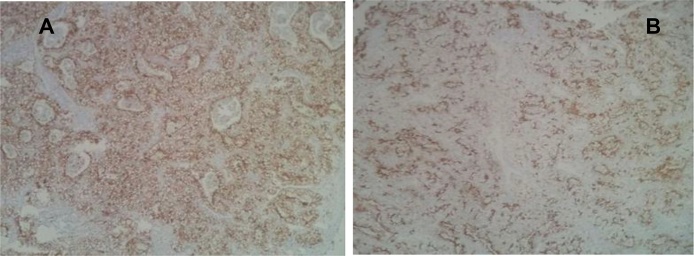
Immunohistochemistry **A.** Neoplastic cells evidencing CD10 immunolabeling positivity. **B**: Neoplastic cells evidencing “Renal Cell Carcinoma” (RCC). immunolabeling positivity.

The anatomopathological examination of the surgical specimen - gallbladder (Fig. 3) was compatible with infiltrating metastasis from clear-cell carcinoma of primary renal site, showing the following markers at immunohistochemistry: vimentin, AE1AE3, CD10, RCC and Racemase-focal (Fig. 4, Fig. 5A and B). At EUS, a solid, hypoechoic, homogeneous, oval nodule with 14 mm was found, with hypoechoic halo in the body region of the pancreas, in the projection of splenomesenteric confluence, next to the splenic vein. Puncture of the lesion was conducted, which cytology was suggestive of clear-cell carcinoma. Because this is an indolent disease with oligometastasis, local ablative treatment with fractionated stereotactic radiation therapy with a dose of 40 Gy was selected. The patient has stable disease one year after radiation therapy.

IMAGE 1A: Expansive formation on the right lateral body wall of the gallbladder, with 1.7 × 1.3 cm, showing pronounced early and persistent contrast enhancement and promoting exophytic bulging of the underlying outer vesicular margin, which shows irregular contours (Red circle). 1B: T2-weighted hypointense expansive formation in the right lateral body wall of the gallbladder (Yellow circle) and T2-weighted slightly hyperintense nodular formation in the body portion of the pancreas (White circle).

IMAGE 2: T1-weighted hypointense nodular formation in the body portion of the pancreas with 1.5 × 1.2 cm (circle).

IMAGE 3: Metastatic renal cell carcinoma as a well-circumscribed polypoid mass in the gallbladder body (circle).

IMAGE 4: Gallbladder with areas of mucosal erosion (arrow) and metastatic neoplastic process constituted of wide and clear cytoplasm cells, permeating the wall of the organ (circle).

IMAGE 5: Immunohistochemistry A: Neoplastic cells evidencing CD10 immunolabeling positivity. B:Neoplastic cells evidencing “Renal Cell Carcinoma” (RCC) immunolabeling positivity.

## 3 Discussion

RCC is one the most lethal tumors of the urological system. Its five-year survival rate in all stages is approximately 69% [2]. This is a tumor of high metastatic potential, whether synchronous or metachronous, and the main distant lesion sites by frequency are: lungs, bones, liver, lymph nodes, adrenal glands, and brain [2,6]. One third of the cases already present with synchronous metastasis upon diagnosis. Other 30% will develop metachronous disease, with 10% with late diagnosis, sometimes ten years post-nephrectomy [7,8].

GB tumors are often diagnosed as polypoid masses and have a wide range of differential diagnoses. The most common are primary vesicular adenomas and adenocarcinomas. Metastases in this organ are rare events in the clinical practice, with primary stomach tumors, melanomas and RCC being the main origins [8].

CT is the most common imaging method in oncology for planning the therapeutic strategy; however, even with this tool, differentiation between primary vesicular tumors and metastatic lesions is still difficult [2,9], with biopsy combined with immunohistochemistry being required for confirmation of diagnosis. In primary tumors, increased CEA and CK7 levels and moderately increased CK10 levels are found. In cases of RCC metastasis, high levels of vimentin are found, with negative CL7 results [2].

There are about 50 RCC metastasis to the gallbladder reported in the literature, from 1963 to the present day, but a significant part of them was diagnosed at autopsy [3,7,10,11] therefore without description of the diagnostic, therapeutic and follow-up process.

Unlike primary gallbladder carcinoma, RCC metastases are predominantly found in male patients, with low incidence of gallstones association [7]. Most cases present as polypoid or pedunculated lesion, as the case reported herein. The diagnosis is usually made accidentally in follow-up examinations, and patients are asymptomatic. Clear Cells was the responsible for almost all cases of metastasis to this organ [7,12].

RCC metastasis to the gallbladder occurs by the systemic dissemination of the disease, not by contiguity, since its appearance is late (average of four years post-nephrectomy [7]), the metastatic lesion is present on the intraluminal surface of the gallbladder, rather than externally (on serosal surface), and there is no predilection of laterality of the RCC – 55% of the GB metastases occur from the right kidney and 45% from the left kidney [9].

A review of RCC metastasis conducted in 2012 evidenced the presence of RCC metastasis to the gallbladder and pancreas in 21% of the patients analyzed, even surpassing lung metastasis [9]. Lung is usually the site of highest rate of metastasis from RCC, with about 60% [13]. Nevertheless, this association was not observed in a review of pancreatic metastasis from RCC, where only one out of 72 patients had such association [14]. In our report, pancreatic metastasis associated with vesicular metastasis has also been diagnosed.

A systematic review in 2015 defined a follow-up algorithm for gallbladder polyps. Based on the risk of malignancy found, polyps greater than 10 mm should be submitted to a surgical procedure, polyps smaller than 4 mm can be observed, with follow up every 2 or 3 years, and polyps between 4 and 10 mm should have their probability of malignancy calculated based on the following criteria: single polyp, sessile polyp and patient age. Polyps with a probability of malignancy greater than 18% should also be resected [15]. In the context of patients with history of RCC, RCC metastasis to the GB should be considered.

## 4 Conclusion

Gallbladder is an unusual site of RCC metastasis; therefore, its diagnosis deserves no active investigation in patients with history of the disease. However, all vesicular lesions in this population should be given attention and surgical treatment should be considered, regardless of the size of the lesion, even when the primary tumor has been treated many years before.

## Conflicts of interest

No conflict of interest relevant to this article was reported.

## Sources of funding

At our own expenses.

## Ethical approval

Ethics approval: Santa Casa de São Paulo Ethics and Research Comittee in 05/11/2018.

Reference number**:** 01571818.5.0000.5479.

## Consent

Written informed consent was obtained from the patient for publication of this case report and accompanying images. A copy of the written consent is available for review by the Editor-in-Chief of this journal on request.

## Author contribution

Mauricio A. Ribeiro: Conceptualization and Methodology; Bruno H. Lucia and Fabio Kater: Investigation, Data collection; Fabiana Bueno: Resources; Maria Carolina Galli Mortati: Writing – Original Draft; Caroline Petersen da C. Ferreira: Writing – Review & Editing and Visualization; Luiz A. Szutan: Supervision and Project Administration.

## Registration of research studies

Case reports that are not first-in-man study already approved in Ethics Committee.

## Guarantor

Caroline Petersen da Costa Ferreira.

## Provenance and peer review

Not commissioned, externally peer-reviewed.
